# Fluoro-2-Deoxyglucose (FDG)-Avid Adenoid Cystic Carcinoma of the Larynx: A Rare Case and Diagnostic Insight Obtained Using Positron Emission Tomography/Computed Tomography (PET/CT) Imaging

**DOI:** 10.7759/cureus.78816

**Published:** 2025-02-10

**Authors:** Serin Moghrabi, Ahmad Al-Muhtaseb, Marwa Y Alshatti, Akram Al-Ibraheem

**Affiliations:** 1 Nuclear Medicine, King Hussein Cancer Center (KHCC), Amman, JOR; 2 Pathology, King Hussein Cancer Center (KHCC), Amman, JOR

**Keywords:** adenoid cystic carcinoma, fdg pet/ct, invasion, larynx, metastases

## Abstract

Adenoid cystic carcinoma (ACC) is a relatively uncommon tumor among head and neck cancers, with laryngeal involvement, typically subglottic, being exceptionally rare. While ACC usually originates in the salivary glands, its occurrence in the larynx is highly unusual. Laryngeal adenoid cystic carcinoma (LACC) is characterized by indolent growth, frequent perineural invasion, and a tendency for local recurrence. Moreover, distant metastasis can occur with the lungs being the most common site of metastasis. We present a case of a 28-year-old male patient with a progressively enlarging right neck mass, uncovering a rare case of infiltrative LACC invading the thyroid gland. Post thyroidectomy, the 18-fluoro-2-deoxyglucose (F-18-FDG)-positron emission tomography (PET)/computed tomography (CT) scan revealed FDG-avid right posterior subglottic residual disease with invasion into adjacent structures, including the trachea. This represents one of the few documented cases of FDG-avid LACC, contributing to the sparse literature on this rare and aggressive malignancy. This case highlights the diagnostic challenge and clinical significance of FDG-PET/CT in evaluating rare and aggressive malignancies like LACC.

## Introduction

Adenoid cystic carcinoma (ACC) is a rare malignancy that primarily originates in the salivary glands, comprising 1-2% of all head and neck cancers and approximately 10% of salivary gland tumors [1]. It most frequently occurs in individuals aged 50 to 60 years and shows no gender predilection [2]. Although ACC primarily arises in the head and neck, it has also been reported in less common sites, such as the larynx, trachea, and lungs, where it originates from submucosal seromucous glands [3]. The occurrence of laryngeal adenoid cystic carcinoma (LACC) is rare and accounts for less than 1% of all malignant tumors occurring in the larynx [4].

Although ACC develops slowly, it is regarded as an aggressive tumor with a high propensity for invading surrounding structures leading to a poor prognosis and necessitating specialized multidisciplinary management [5]. Hematogenous spread is significantly more common than lymphatic spread, with the lungs being the most frequent site of metastasis, followed by the bones, liver, and occasionally the brain [6]. Lymph node involvement is rare compared to other malignancies but, when present, typically involves regional nodes near the primary site [7].

LACC presents a significant diagnostic challenge due to its complex presentation and potential for local invasion and distant metastases [8]. Accurate diagnosis requires a comprehensive multimodality imaging approach, including computed tomography (CT) for detailed assessment of bone and soft tissue involvement, magnetic resonance imaging (MRI) for superior soft tissue contrast and evaluation of perineural spread, and 18-fluoro-2-deoxyglucose (F-18-FDG)-positron emission (PET)/computed tomography (CT) for detecting metabolic activity, identifying distant metastases, and guiding treatment planning [8].

This case is particularly unique due to the presence of ACC in the subglottic region with local extension to the thyroid gland, subsequent tumor recurrence, and detection of a few hypermetabolic regional and distant lymph nodes on FDG-PET/CT.

## Case presentation

**Figure 1:**
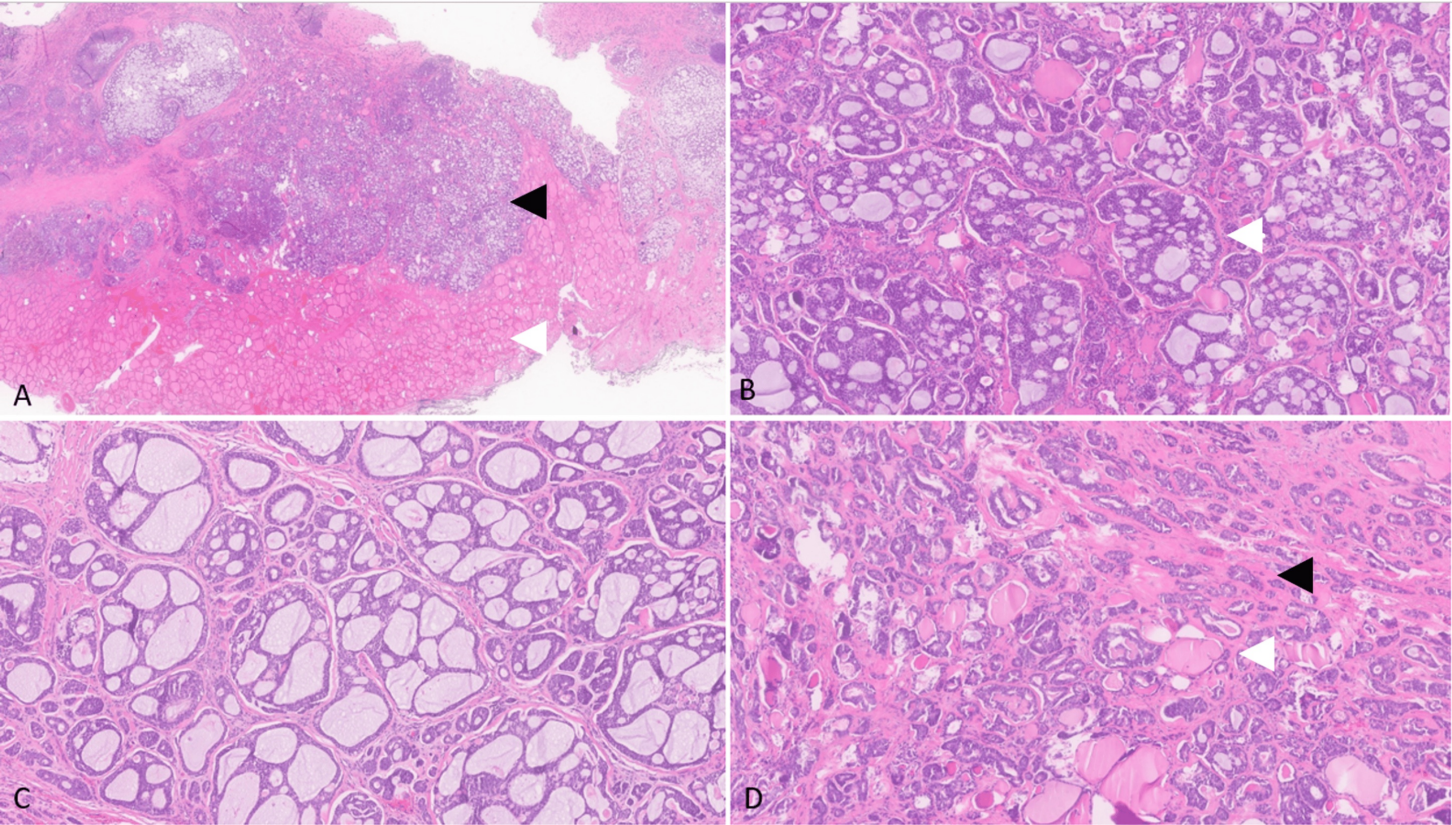
Post-operation histopathology slides (A) Thyroid gland (white arrowhead) infiltration by multiple nodules of tumor cells (black arrowhead). (B) The tumor nodules show the characteristic histopathology of the cribriform pattern of adenoid cystic carcinoma (white arrowhead). (C) There are two types of neoplastic cells: inner epithelial cells and outer multilayered myoepithelial cells. (D) Other tumor foci show tubular patterns of adenoid cystic carcinoma (black arrowhead) invading between thyroid follicles (white arrowhead).

In July 2024, a 28-year-old male patient with no significant past medical or family history presented with a gradually enlarging anterior right neck lump that had been noticeable for the past nine months and became more prominent in the last five weeks. He denied experiencing palpitations, tremors, thermal sensitivity disturbances, nervousness, insomnia, ocular manifestations, weight loss, odynophagia, dysphagia, nasal regurgitation, hoarseness of voice/stridor, or alterations in bowel habits. Upon physical examination, a well-defined nodule located in the right thyroid lobe was noted. The nodule measured approximately 2x2 cm in diameter and appeared to be solid in consistency. It was non-tender to palpation and felt fixed to the surrounding thyroid tissue. Moreover, his thyroid function and other laboratory tests were unremarkable. A subsequent neck ultrasound revealed the presence of a substantial right inferior thyroid nodule measuring about 2x2 cm and extending into the retrosternal region. This nodule exhibited heterogeneity, internal vascularity, and cystic changes. There was no evidence of enlarged cervical lymph nodes. Fine-needle aspiration was not inconclusive. Thus, surgical intervention was decided, and he underwent a total thyroidectomy in August 2024, which revealed a 2 x 1.8 x 1 cm tumor in the right thyroid lobe. On histopathological evaluation, the immunohistochemical analysis showed that tumor cells exhibited positive staining for cytokeratin 7 (CK7), lymphoid enhancer-binding factor 1 (LEF1), calponin, and s100 (focal) while negative for GATA3 and TTFl. Notably, B-catenin showed positive membranous staining. Due to the pathology and immunohistochemistry analysis, the diagnosis of ACC was confirmed to be consistent with a high-grade malignant ACC involving the thyroid parenchyma T4aNxMx (Figure 1).

Then, the patient was referred to a tertiary hospital for comprehensive assessment and treatment, which included a whole-body CT showing a right posterior subglottic residual mass invading the trachea, associated with bilateral neck lymph nodes, a small right basal pleural-based lower lobe pulmonary nodule, and bilateral axillary, external iliac, and inguinal lymph nodes.

Subsequent bronchoscopy detected a subglottic tumor infiltrating the tracheal mucosa (sparing the left side) and causing focal narrowing by more than 50%, with a polypoid component in the anterior right aspect of the trachea. The involved area was about 1-1.5 cm below the vocal cords, 4 cm in length, and about 8 cm above the main carina.

**Figure 2:**
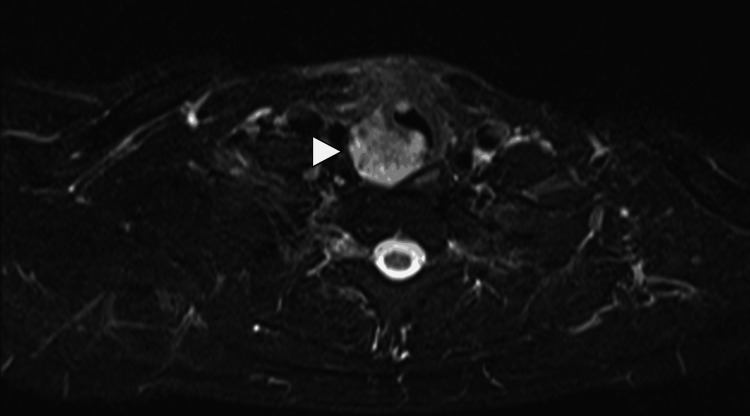
MRI neck with contrast The short Tau inversion recovery magnetic resonance imaging (STIR MRI) image shows a hyperintense contrast-enhancing soft tissue lesion at the right upper para and retro tracheal area extending slightly to the left side.

**Figure 3:**
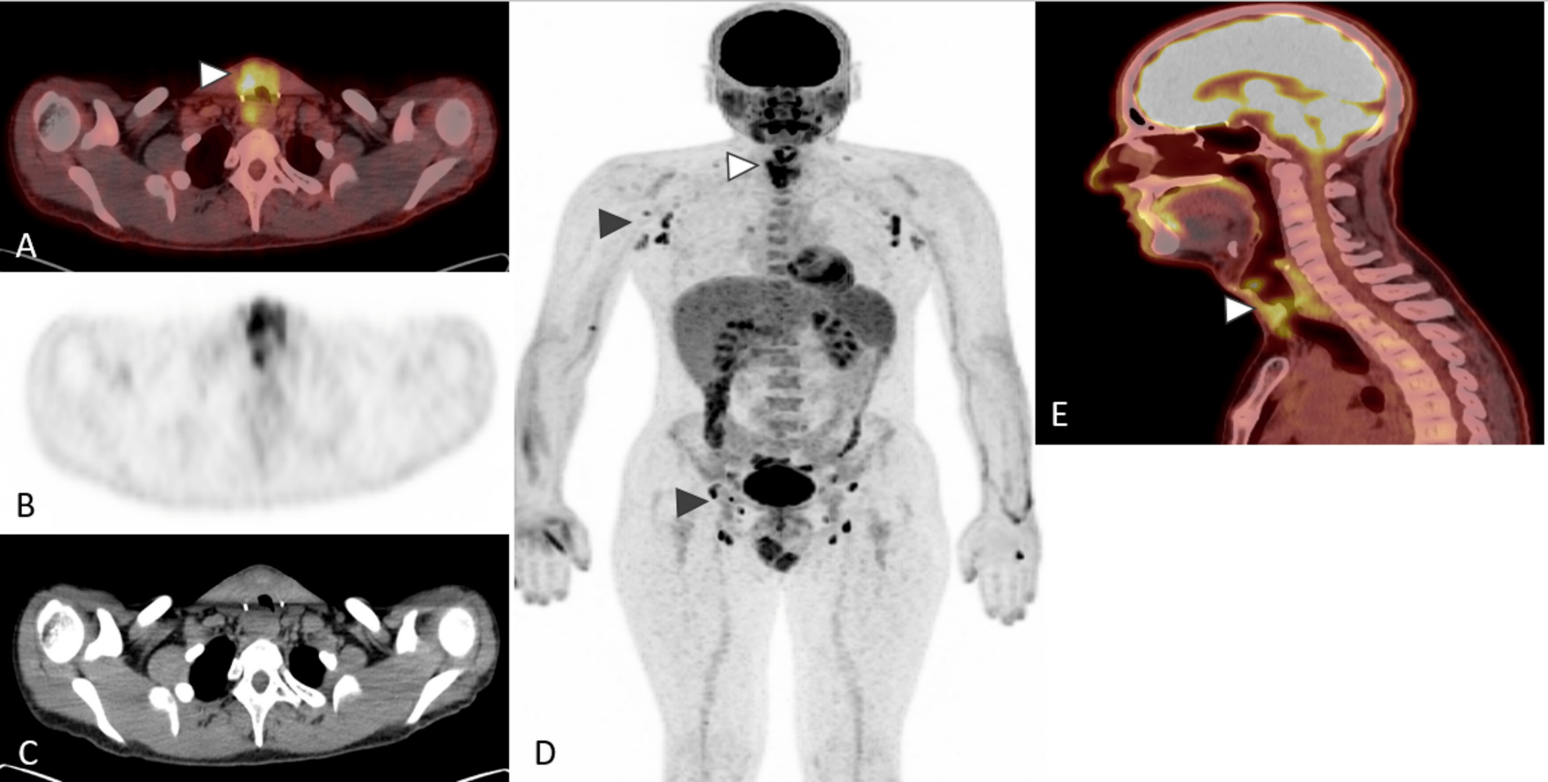
PET/CT images Axial fused PET/CT images (A, B, C) demonstrating, by the white arrowhead, a hypermetabolic right posterior subglottic residual mass invading the trachea (SUVmax = 8.6). (D) Maximum intensity projection (MIP) image demonstrates a hypermetabolic right posterior subglottic residual mass invading the trachea (maximum standardized uptake value (SUVmax) = 8.6) demonstrated by the white arrowhead, hypermetabolic metastatic bilateral cervical lymph nodes (SUVmax = 4.6), and multiple hypermetabolic bilateral axillary, external iliac, and bilateral inguinal lymph nodes, demonstrated by the two black arrowheads (SUVmax = 7.1). Sagittal fused PET/CT image (E) shows the subglottic lesion invading the adjacent trachea, demonstrated by the white arrowhead.

A neck MRI showed residual nodules in the posterior right subglottic area, measuring about 1.9 cm, infiltrating the adjacent trachea, with small bilateral cervical lymph nodes measuring up to 1.0 cm (Figure 2). FDG-PET/CT was ordered for better evaluation of the aforementioned lesions, and it showed a hypermetabolic posterior right subglottic residual mass invading the trachea (maximum standardized uptake value (SUVmax) = 8.6), hypermetabolic metastatic bilateral cervical lymph nodes (SUVmax = 4.6), and multiple hypermetabolic bilateral axillary, external iliac, and bilateral inguinal lymph nodes (SUVmax = 7.1), which are unusual for laryngeal tumor metastases (Figure 3). Tissue confirmation was done for the right posterior subglottic residual mass, indicating LACC. Also, a tissue confirmation from the inguinal lymph nodes showed reactive lymphoid tissue, making the TNM staging T4aN2M0.

The multidisciplinary team discussed the case, initially considering surgery with tumor resection and anastomosis. Due to the tumor's size, a laryngectomy with a permanent tracheostomy was also an option, but the patient declined surgery after reviewing the risks. Instead, they opted for definitive chemoradiation, with regular follow-ups planned to monitor progress.

## Discussion

ACC most commonly originates in the salivary glands, with a distinct preference for minor salivary glands. These glands are located across various anatomical regions, including the oral cavity, palate, and upper respiratory tract. However, LACC is extremely rare owing to the scarcity of salivary glands in the mucosa of the larynx and trachea [9,10]. 

The rarity of LACC is influenced by the distribution of submucosal glands, primarily concentrated in the subglottic region [9,10]. The symptoms associated with LACC can vary based on its location; however, they are often atypical and may not manifest until the tumor has advanced significantly [9], such as in this case report, the tumor showed no typical symptoms until it progressed to infiltrate the surrounding structures like the thyroid gland forming a nodule that prompted medical evaluation. Due to its low incidence, atypical symptoms, and delayed onset of clinical signs, diagnosing LACC can be particularly difficult. The delayed onset of clinical signs often leads to advanced-stage presentations by the time of diagnosis [4]. Moreover, LACC is slow-growing and aggressive, manifested by perineural invasion and infiltrative growth; given the aggressive nature of LACC, advanced imaging modalities like FDG-PET/CT can be advantageous in assessing tumor behavior and identifying metastatic sites [11]. Two case reports in the literature have highlighted the utility of FDG-PET in LACC. One report showed that FDG PET/CT helped in an accurate staging and enabled the patient to proceed with surgery to excise the primary tumor, by excluding pulmonary nodule metastases, which were suspicious on the CT scan [12]. The other showed that FDG-PET was truly negative for lymph node metastasis, despite palpable lymph nodes on examination and suspicious findings on MRI. The absence of FDG uptake was later confirmed by biopsy, illustrating FDG-PET/CT is a valuable tool [9].

While the role of FDG-PET /CT in LACC is still being explored, its effectiveness in ACC in general is documented in the literature. It showed high sensitivity in detecting recurrent or residual tumors and regional and distant metastases in patients with ACC [13]. Jung et al. reported a sensitivity of approximately 87% for FDG-PET/CT in detecting primary ACC of the head and neck of the minor salivary, oral cavity, nasal cavity, and paranasal sinuses, also adding that total lesion glycolysis (TLG) was associated with progression-free survival [14]. For recurrence assessment, Ruhlmann et al. reported that the diagnostic performance of PET/CT is comparable to MRI in local restaging with a higher sensitivity (96% v. 89%) and diagnostic accuracy (94% vs. 89%) [13]. It also identified distant metastases including cases where CT missed FDG-positive lesions [13].

FDG-PET/CT is increasingly recognized in ACC for detecting distant metastases and assessing tumor activity, providing valuable prognostic information. Metrics such as SUVmax, metabolic tumor volume (MTV), and TLG are key in evaluating tumor behavior. High SUVmax values, in particular, are indicative of a higher risk of metastasis, especially to the lungs. SUVmax can also predict tumor progression after treatment, while MTV and TLG are useful in predicting the development of distant metastases, both at initial staging and during follow-up [15]. This is particularly relevant for clinicians, as distant metastases often lead to disease-specific death in ACC patients [15]. In addition to the utility of PET/CT scans, in recent years, the emergence of new imaging modalities such as Gallium-68 fibroblast activation protein inhibitor (Ga-68 FAPI) PET/CT has shown promise in further improving ACC staging and treatment planning. A study has found that 68Ga-FAPI PET/CT led to upstaging and identiﬁcation of additional metastases, thereby altering the staging in 42% of cases. Furthermore, it was found that 68Ga-FAPI PET/CT was more accurate in delineating radiotherapy target volumes than CT scans and MRI scans [16]. However, 68Ga-FAPI PET/CT has not yet been tested for LACC. Further research and case reports are needed to evaluate its potential utility in accurately assessing LACC, particularly in terms of tumor staging, treatment planning, and monitoring response to therapy. Our patient, a 28-year-old man, was diagnosed with LACC following a total thyroidectomy. Despite the complete surgical removal of the thyroid, a post-operative FDG-PET/CT scan revealed infiltrative residual disease extending into the trachea, with significant FDG avidity (SUVmax 8.6), indicating the persistence of aggressive tumor activity. Also, it identified enlarged axillary, external iliac, and inguinal lymph nodes, sites rarely associated with ACC metastasis. These findings were unusual for the possibility of metastases, as ACC metastases typically involve the lungs, bones, the liver, and occasionally the brain [6]. Biopsies of these distant lymph nodes confirmed reactive lymphoid tissue. 

Research suggests that a combination of conservative surgery, postoperative radiotherapy, and chemotherapy is critical for minimizing the risk of locoregional recurrence [17]. Achieving complete tumor resection can be challenging, and local recurrences or distant metastases may arise even several years after initial treatment [17]. As a result, ongoing and long-term monitoring is essential to detect any late relapses or metastases [17].

## Conclusions

LACC remains a rare and challenging malignancy, often diagnosed at an advanced stage due to its slow-growing nature and atypical symptoms. This case illustrates the importance of a comprehensive, multimodal imaging approach, including FDG-PET/CT and MRI, to accurately assess tumor behavior and detect residual disease. While the use of FDG-PET/CT in ACC is well-established, further research is needed to explore its full potential in LACC, along with emerging modalities like Ga-68 FAPI PET/CT. Given the aggressive behavior of ACC and LACC, multidisciplinary management involving surgery, chemoradiation, and long-term follow-up is crucial to optimize patient outcomes and monitor for recurrence or metastasis. Ongoing surveillance and early detection remain key in improving survival rates and overall prognosis for patients with this rare tumor.
